# Maternal environmental noise exposure during the periconceptional period and risk of congenital heart disease in offspring: a case-control study in Northwest China

**DOI:** 10.3389/fpubh.2026.1923108

**Published:** 2026-09-01

**Authors:** Mingxin Yan, Yan Zhao, Yunbo Zhang, Doudou Zhao, Bin Wang, Jianting Si, Yuanhan Sun, Guoqiang Dong, Leilei Pei, Pengfei Qu

**Affiliations:** 1 Department of Epidemiology and Biostatistics, School of Public Health, Xi’an Jiaotong University Health Science Center, Xi’an, China; 2 Center for Occupational Safety and Health, Institute for Hygiene of Ordnance Industry, Xi’an, China; 3 Department of Dermatology, The First Affiliated Hospital of Xi’an Jiaotong University, Xi’an, China; 4 Translational Medicine Center, Northwest Women’s and Children’s Hospital, Xi’an, China

**Keywords:** congenital heart disease, environmental epidemiology, environmental noise exposure, periconceptional period, traffic noise

## Abstract

**Background:**

Environmental noise is a pervasive public health concern, yet evidence linking maternal environmental noise exposure during the periconceptional period to the risk of congenital heart disease (CHD) in offspring remains limited, particularly in low and middle-income settings.

**Methods:**

We conducted a multicenter hospital-based case-control study in Xi’an, Northwest China, including 589 mothers of infants with CHD and 1193 frequency-matched controls. Maternal periconceptional noise exposure was assessed using standardized questionnaires, capturing exposure duration, perceived interference, and dominant noise sources (traffic, construction, and industrial). Multivariable logistic regression estimated odds ratios (ORs) and 95% CIs, adjusting for potential confounders. Restricted cubic spline analysis assessed nonlinear exposure–response relationships.

**Results:**

Maternal periconceptional noise exposure was associated with increased CHD risk (OR, 1.81; 95% CI, 1.36–2.40). Compared with no noise exposure, exposure for 4 to ≤8 hours per day was associated with higher risk (OR, 3.24; 95% CI, 2.02–5.20), as was exposure for more than 8 hours per day (OR, 2.07; 95% CI, 1.19–3.58). Traffic noise (OR, 1.75; 95% CI, 1.20–2.56) and construction and industrial noise (OR, 2.13; 95% CI, 1.29–3.50) were also independently associated with increased risk. Restricted cubic spline analysis indicated a positive nonlinear association between noise exposure duration and CHD risk.

**Conclusion:**

Maternal periconceptional exposure to environmental noise was associated with increased CHD risk in offspring, particularly with prolonged exposure and specific sources such as traffic and construction. Reducing environmental noise exposure during the periconceptional period may be important for CHD prevention.

## Introduction

Congenital heart disease (CHD) is the most common type of birth defect and is associated with substantial risks, including miscarriage, stillbirth, and infant mortality (1, 2). Globally, the incidence of CHD is estimated at 4 to 10 cases per 1000 live births and is steadily rising (3). In China, national birth defect surveillance data suggest that approximately 150,000 newborns are diagnosed with CHD annually, corresponding to an incidence of 8 to 10 per 1000 live births (4). The burden may be even higher in low and middle-income countries, where comprehensive epidemiological data are often lacking (5). Although both genetic and environmental factors have been implicated in the etiology of CHD, the precise pathogenic mechanisms remain largely unclear (6).

Amid rapid socioeconomic development and urbanization, environmental noise has emerged as a growing public health concern. Beyond its well-documented effects on the auditory system, noise exposure has been associated with a range of adverse physical and mental health outcomes, including cardiovascular and metabolic disorders, as well as sleep disturbances (7, 8). Increasing evidence suggests that prenatal exposure to environmental noise may contribute to adverse birth outcomes such as low birth weight, preterm birth, and small-for-gestational-age status (9–11). Notably, residential outdoor noise exposure during pregnancy has also been associated with altered embryonic growth (12). Experimental studies in animal models have further demonstrated the teratogenic potential of noise exposure, with increased rates of congenital anomalies and perinatal mortality observed in rodents (13–15). Despite these findings, direct evidence linking maternal environmental noise exposure during the periconceptional period to the risk of congenital heart disease in offspring remains limited.

In this study, we examined the association between maternal environmental noise exposure characteristics—including exposure duration and sources—and the risk of CHD using data from a multicenter, hospital-based case-control study. We further assessed the associations between specific noise sources, including traffic and construction-related noise, during the periconceptional period and CHD risk in offspring.

## Subjects and methods

### Study design and participants

This study was based on a case–control investigation of risk factors for CHD, conducted from January 2014 to December 2016 across six tertiary hospitals in Xi’an, Shaanxi Province, China. The study protocol was approved by the Biomedical Ethics Committee of the Department of Medicine, Xi’an Jiaotong University (Approval No. 2012008). All procedures involving human participants adhered to the ethical standards of the Declaration of Helsinki. Written informed consent was obtained from all participants prior to enrollment.

The study population included mothers of fetuses diagnosed with CHD (case group) and mothers of fetuses without CHD (control group). Inclusion criteria for the case group were: (1) pregnancy termination or delivery between January 2014 and December 2016; and (2) a CHD diagnosis confirmed by a pediatric cardiologist based on the International Classification of Diseases, 10th Revision (ICD-10), and fetal echocardiography performed between the 28th week of gestation and the 7th day postpartum. Fetuses with congenital anomalies unrelated to CHD were excluded.

For each case, two controls were selected. The inclusion criteria for controls were: selection from the same hospital as the corresponding case; pregnancy ending in the same calendar year; absence of genetic disorders or birth defects in the fetus; and the parents’ ability to complete the questionnaire reliably. Parents with psychiatric disorders or severe illnesses that impaired their ability to provide accurate responses were excluded.

**Figure 1:**
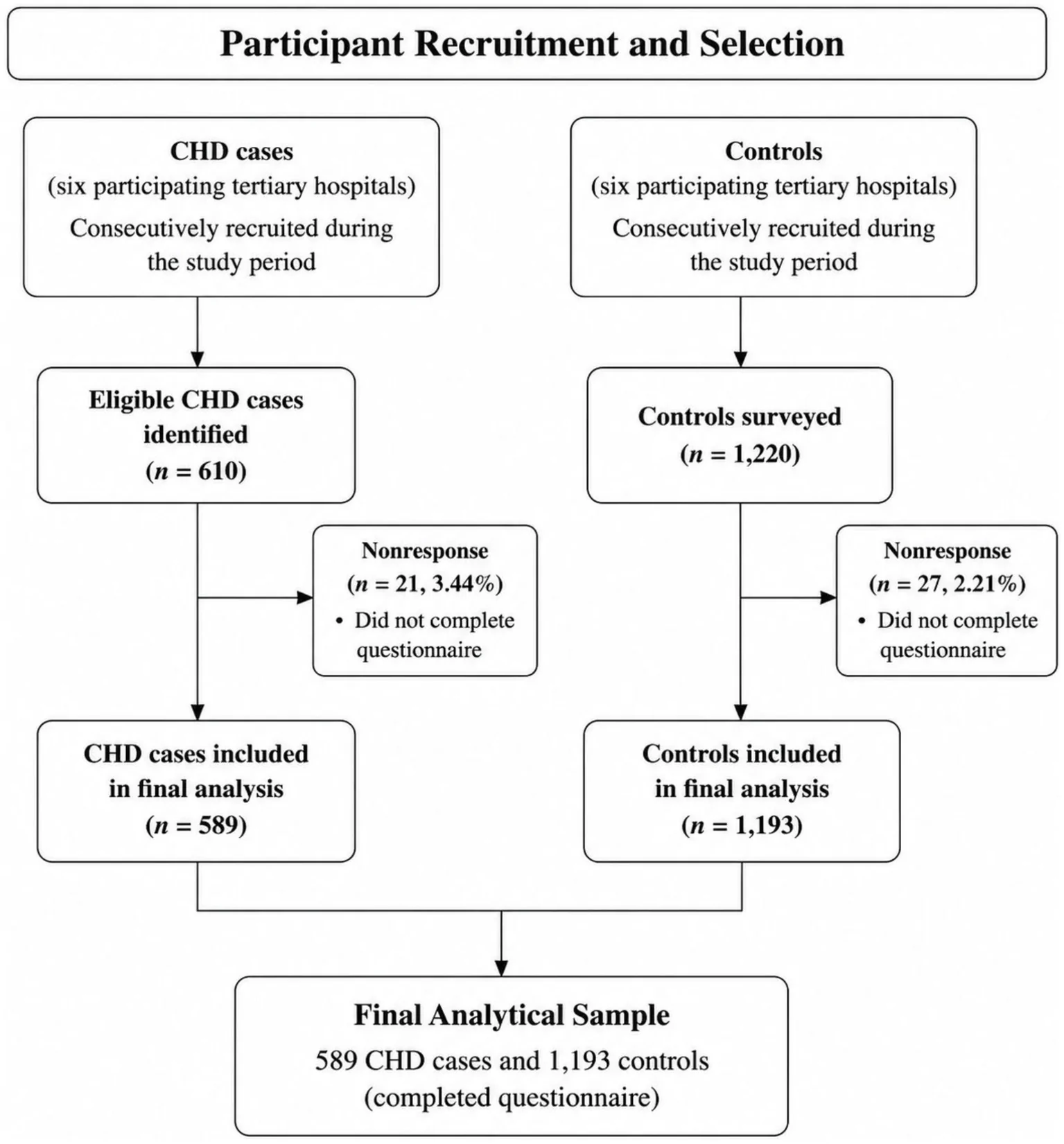
Flow diagram of participant recruitment and inclusion in the study.

Eligible CHD cases and controls were consecutively recruited from the six participating tertiary hospitals during the study period. In the case group, 610 participants were initially recruited, with a nonresponse rate of 3.44% (21 individuals). In the control group, 1220 participants were surveyed, with a nonresponse rate of 2.21% (27 individuals). Ultimately, 589 cases and 1193 controls who completed the questionnaire were included in the final analysis (Figure 1).

### Data collection

Trained postgraduate students from the Faculty of Medicine at Xi’an Jiaotong University conducted face-to-face interviews using standardized, structured questionnaires. The questionnaire was developed based on previous epidemiological studies assessing maternal environmental exposures and according to the classification of environmental noise sources specified in Chinese national environmental noise standards. These questionnaires collected information on sociodemographic characteristics, lifestyle behaviors, and nutritional supplementation during pregnancy, medical history and medication use, family history of birth defects, and exposure to environmental risk factors.

Before formal data collection, a pilot survey was conducted to assess the feasibility, clarity, and comprehensibility of the questionnaire items. Based on feedback from participants and investigators, the wording and sequence of questions were refined to improve understanding and response accuracy. All interviewers received standardized training before the investigation, and structured face-to-face interviews were conducted using uniform procedures to minimize information bias. When necessary, family members were also interviewed to enhance the accuracy of recalled information.

Information on neonatal outcomes and pregnancy diagnoses was supplemented by reviewing hospital medical records. Medical records were also reviewed to identify relevant maternal medical conditions, including documented hearing-related disorders, and no participants with recorded hearing impairment were identified. All clinical diagnoses were further reviewed and confirmed by an expert panel comprising senior clinicians, including cardiovascular epidemiologists, obstetricians, pediatricians, and radiologists from Northwest Women’s and Children’s Hospital. Additionally, a follow-up telephone interview was conducted within one year postpartum to validate clinical diagnoses.

### Definition of noise exposure

Information on maternal environmental noise exposure during the periconceptional period—including exposure duration, noise sources, and residential proximity to major roads traffic and construction sites—was collected through self-reported questionnaires administered to participating mothers. In this study, noise exposure was defined as environmental sound perceived by participants as interfering with their ability to rest, study, or work during the periconceptional period, including the period before conception and early pregnancy (16). This exposure window was selected because pregnancy recognition generally occurs after conception, and exposures before pregnancy awareness may coincide with early embryonic development, including the period of cardiac morphogenesis. The exposure assessment was based on perceived noise interference, reflecting the functional impact of environmental noise on daily activities rather than a direct measurement of physical noise intensity or a standardized assessment of psychological noise annoyance.

The questionnaire assessed noise exposure based on three dimensions: exposure status (yes/no), daily exposure duration (hours/day), and major exposure sources. Daily exposure duration was categorized according to predefined intervals (0, >0 to ≤4, >4 to ≤8, and >8 hours/day) to facilitate statistical analysis. Noise sources were classified according to China’s national environmental standards into four main categories: transportation, construction, industrial production, and social activity (17).

Transportation noise referred to disturbances from motor vehicles, railway systems, urban rail transit, and other traffic operations affecting the residential environment. Construction noise included sounds generated by activities such as subway development, residential building, and renovation work. Industrial production noise encompassed noise from manufacturing processes that disrupted nearby residential areas. Social activity noise referred to disturbances from human activities, including school environments, neighborhood gatherings, and recreational events (eg, public square dancing).

Because cases involving multiple simultaneous noise sources were rare, analyses focused on the most frequently reported single source to improve interpretability. In addition, due to the small number of participants exposed to industrial production noise (*n* = 9), this category was combined with construction noise for analysis.

In addition, residential proximity to major roadways and construction sites was also considered a proxy for environmental noise exposure. Participants were asked to self-report whether their residence was located near major roadways or construction sites and to estimate the approximate distance. Although objective noise measurements were unavailable, these residential proximity indicators provided additional information related to potential environmental noise sources and were analyzed separately to evaluate the robustness of the association. The distance from the participant’s residence to the nearest major road was classified into four categories: ≤50 meters, >50 to ≤100 meters, >100 to ≤300 meters, and >300 meters. Distances to construction sites were grouped into three categories: ≤500 meters, >500 to ≤1000 meters, and >1000 meters.

### Definition of study outcomes

The primary outcome in this study was congenital heart disease (CHD). Diagnoses were confirmed by specialized physicians at the participating hospitals based on a comprehensive assessment of medical history, clinical presentation, physical examination findings, and diagnostic imaging. The CHD subtypes considered in this study included ventricular septal defect (VSD), atrial septal defect (ASD), patent ductus arteriosus (PDA), atrioventricular septal defect (AVSD), and tetralogy of Fallot (TOF), among others (18).

### Covariates

Building on previous research (19, 20), data on potential confounding variables associated with congenital heart disease (CHD) were also collected for study participants. These variables included sociodemographic characteristics, lifestyle behaviors, and health-related factors. Sociodemographic characteristics included age of pregnant women (<30 years and ≥30 years), ethnicity (Han and other), residence (urban and rural), education (senior high school or lower and college or above), and family wealth index (poor, moderate, and rich). Lifestyle behaviors included fever in early pregnancy (no, yes), cold in early pregnancy (no, yes), taking medicine (no, yes), passive smoking during pregnancy (no, yes), alcohol consumption during pregnancy (no, yes), hair dyeing during pregnancy (no, yes), and cooking during pregnancy (no, yes). Health-related characteristics included folic acid supplementation (no, yes), family history of birth defects (no, yes), and frequency of pregnancy (1, ≥2).

The family wealth index was constructed using principal component analysis, incorporating variables such as monthly expenditure, monthly household income, household appliances, type of housing and transportation (21). Passive smoking during pregnancy is defined as inhaling smoke for more than 15 minutes per day for one month or longer during pregnancy. Alcohol consumption during pregnancy is defined as the consumption of alcoholic products (liquor, beer, wine, etc.) on one or more occasions during pregnancy. Hair coloring and perming during pregnancy entail engaging in one or more instances of hair coloring or perming during pregnancy. Taking folic acid during pregnancy is defined as the consistent intake of folic acid for one month or more, spanning from three months before pregnancy to the pregnancy period.

### Statistical analysis

Categorical variables were presented as frequencies (n) and percentages (%), and group comparisons were conducted using the χ^2^ test. Non-normally distributed continuous variables were expressed as median (25th–75th percentile), and their group comparisons were performed using the Wilcoxon rank test.

The impact of noise exposure on CHD and its major subtypes (VSD, ASD, PDA, AVSD, and TOF) was evaluated using univariate and multivariate logistic regression models Model 2 was adjusted for age, ethnicity, residence, education, and family wealth index. Model 3 was further adjusted for additional covariates, including fever and cold in early pregnancy, medication use, parity, passive smoking, alcohol consumption, hair dyeing or perming, cooking frequency, folic acid supplementation, and family history of birth defects. For analyses of specific CHD subtypes, Bonferroni correction was applied to account for multiple comparisons, with a corrected significance threshold of 0.01.

Subgroup logistic regression analyses were performed to evaluate the effects of noise exposure duration and source on CHD risk. Restricted cubic spline (RCS) regression was applied to explore potential nonlinear relationships between daily noise exposure duration and the incidence of CHD. In addition, residential proximity to major roads and construction sites was analyzed as a proxy for environmental noise exposure to further assess the effects of road traffic and construction noise on CHD risk.

To test the robustness of our findings, several sensitivity analyses were conducted. First, we performed stratified analyses to explore potential heterogeneity in the associations between noise exposure and CHD across subgroups defined by maternal characteristics. Second, propensity score matching was used to control for confounding and to further validate the association between noise exposure and CHD.

All statistical analyses were performed using SAS version 9.4 (SAS Institute, Cary, NC, United States). A two-sided *P* value <0.05 was considered statistically significant.

## Results

### Participants’ characteristics

A total of 589 cases and 1,193 controls were included in the final analysis. Table 1 presents the baseline characteristics of participants in the case and control groups. Compared with the control group, mothers in the case group were younger, had lower educational attainment and family wealth index, and were less likely to be of Han ethnicity. They also exhibited higher prevalence of early-pregnancy cold, medication use, gravidity ≥2, passive smoking, alcohol consumption, hair dyeing or perming, cooking during pregnancy, and family history of birth defects, as well as lower rates of folic acid supplementation. All differences were statistically significant (*P* < 0.05).

**Table 1:** Baseline characteristics of the case and control groups.

Variables	Cases (*N* = 589)	Controls (*N* = 1193)	*χ^2^/Z*	*P* value
Age, median (IQR)	26.93 (24.21, 30.72)	28.60 (26.33, 31.42)	−6.406	<0.001^a^
Ethnicity, *n* (%)			10.894	0.001
Han	574 (97.45)	1185 (99.33)		
Other	15 (2.55)	8 (0.67)		
Residence, *n* (%)			178.734	<0.001
Urban	196 (33.28)	796 (66.72)		
Rural	393 (66.72)	397 (33.28)		
Education, *n* (%)			88.602	<0.001
Senior high school or lower	261 (44.31)	270 (22.63)		
College or above	328 (55.69)	923 (77.37)		
Family wealth index, *n* (%)			117.748	<0.001
Poor	308 (52.29)	328 (27.49)		
Moderate	165 (28.01)	395 (33.11)		
Rich	116 (19.70)	470 (39.40)		
Fever in early pregnancy, *n* (%)			3.438	0.064
No	535 (90.83)	1113 (93.29)		
Yes	54 (9.17)	80 (6.71)		
Cold in early pregnancy, *n* (%)			22.329	<0.001
No	406 (68.93)	944 (79.13)		
Yes	183 (31.07)	249 (20.87)		
Taking medicine, *n* (%)			17.359	<0.001
No	460 (78.10)	1025 (85.92)		
Yes	129 (21.90)	168 (14.08)		
Gravidity, *n* (%)			25.378	<0.001
1	230 (39.05)	617 (51.72)		
≥2	359 (60.95)	576 (48.28)		
Passive smoke, *n* (%)			38.308	<0.001
No	248 (42.11)	688 (57.67)		
Yes	341 (57.89)	505 (42.33)		
Drinking, *n* (%)			12.422	<0.001
No	573 (97.28)	1185 (99.33)		
Yes	16 (2.72)	8 (0.67)		
Hair dyeing and perming, *n* (%)			12.638	<0.001
No	553 (93.89)	1161 (97.32)		
Yes	36 (6.11)	32 (2.68)		
Cooking, *n* (%)			12.093	<0.001
No	166 (28.18)	435 (36.46)		
Yes	423 (71.82)	758 (63.54)		
Folic acid supplement, *n* (%)			20.680	<0.001
No	122 (20.71)	149 (12.49)		
Yes	467 (79.29)	1044 (87.51)		
Family history of birth defects, *n* (%)			13.282	<0.001
No	530 (89.98)	1129 (94.64)		
Yes	59 (10.02)	64 (5.36)		

IQR, interquartile range.

^a^Wilcoxon rank test.

### Noise exposure and risk of CHD

The prevalence of maternal noise exposure during the periconceptional period was higher in the case group than in controls (23.4% vs 13.9%) (Table 2). After adjusting for potential confounders, noise exposure was associated with an increased risk of CHD in offspring (adjusted OR, 1.81; 95% CI; 1.36–2.40). Further analyses explored the association between noise exposure and specific CHD subtypes (Supplementary Table S1). Among the CHD subtypes examined, the association with TOF remained statistically significant after Bonferroni correction (adjusted OR, 2.85; 95% CI; 1.46–5.56). No statistically significant associations were observed for other CHD subtypes.

**Table 2:** Association between noise exposure and CHD.

Variables	Cases *N* (%)	Controls *N* (%)	Model 1 crude OR (95% CI), *P* value	Model 2^a^ adjusted OR (95% CI), *P* value	Model 3^b^ adjusted OR (95% CI), *P* value
Noise exposure					
No	451 (76.57)	1027 (86.09)	1.00	1.00	1.00
Yes	138 (23.43)	166 (13.91)	1.89 (1.47, 2.43), <0.001	1.99 (1.51, 2.61), <0.001	1.81 (1.36, 2.40), <0.001
Noise exposure duration (continuous, per 1-hour/day increase)	—	—	1.06 (1.03, 1.09), <0.001	1.07 (1.04, 1.11), <0.001	1.06 (1.03, 1.09), <0.001
Noise exposure duration (hours/day)					
0	451 (76.57)	1027 (86.09)	1.00	1.00	1.00
0 ~ ≤4	51 (8.66)	91 (7.63)	1.28 (0.89, 1.83), 0.185	1.23 (0.83, 1.81), 0.305	1.14 (0.76, 1.70), 0.528
4 ~ ≤8	55 (9.34)	40 (3.35)	3.13 (2.05, 4.78), <0.001	3.58 (2.26, 5.68), <0.001	3.24 (2.02, 5.20), <0.001
>8	32 (5.43)	35 (2.93)	2.08 (1.27, 3.41), 0.003	2.37(1.39, 4.03), 0.001	2.07 (1.19, 3.58), 0.010
Noise exposure sources					
No	451 (76.57)	1027 (86.09)	1.00	1.00	1.00
Transportation	66 (11.21)	82 (6.87)	1.83 (1.30, 2.58), <0.001	1.87 (1.29, 2.70), <0.001	1.75 (1.20, 2.56), 0.004
Construction, industrial production	39 (6.62)	43 (3.60)	2.07 (1.32, 3.23), 0.001	2.37 (1.46, 3.84), <0.001	2.13 (1.29, 3.50), 0.003
Social life	33 (5.60)	41 (3.44)	1.83 (1.14, 2.94), 0.012	1.85 (1.11, 3.09), 0.018	1.61 (0.94, 2.75), 0.081

For continuous exposure analysis, noise exposure duration was modeled as a continuous variable; the OR represents the change in odds of CHD per 1-hour/day increase in exposure duration.

Noise exposure sources: Transportation (motor vehicles, railroad rolling stock, urban rail transit vehicles, etc.).

^a^Model 2 adjusted age, ethnicity, residence, education, and family wealth index.

^b^Model 3 adjusted age, ethnicity, residence, education, family wealth index, fever in early pregnancy, cold in early pregnancy, taking medicine, gravidity, passive smoke, drinking, hair dyeing and perming, cooking, folic acid supplement, and family history of birth defects.

### Duration and sources of noise exposure in relation to CHD

**Figure 2:**
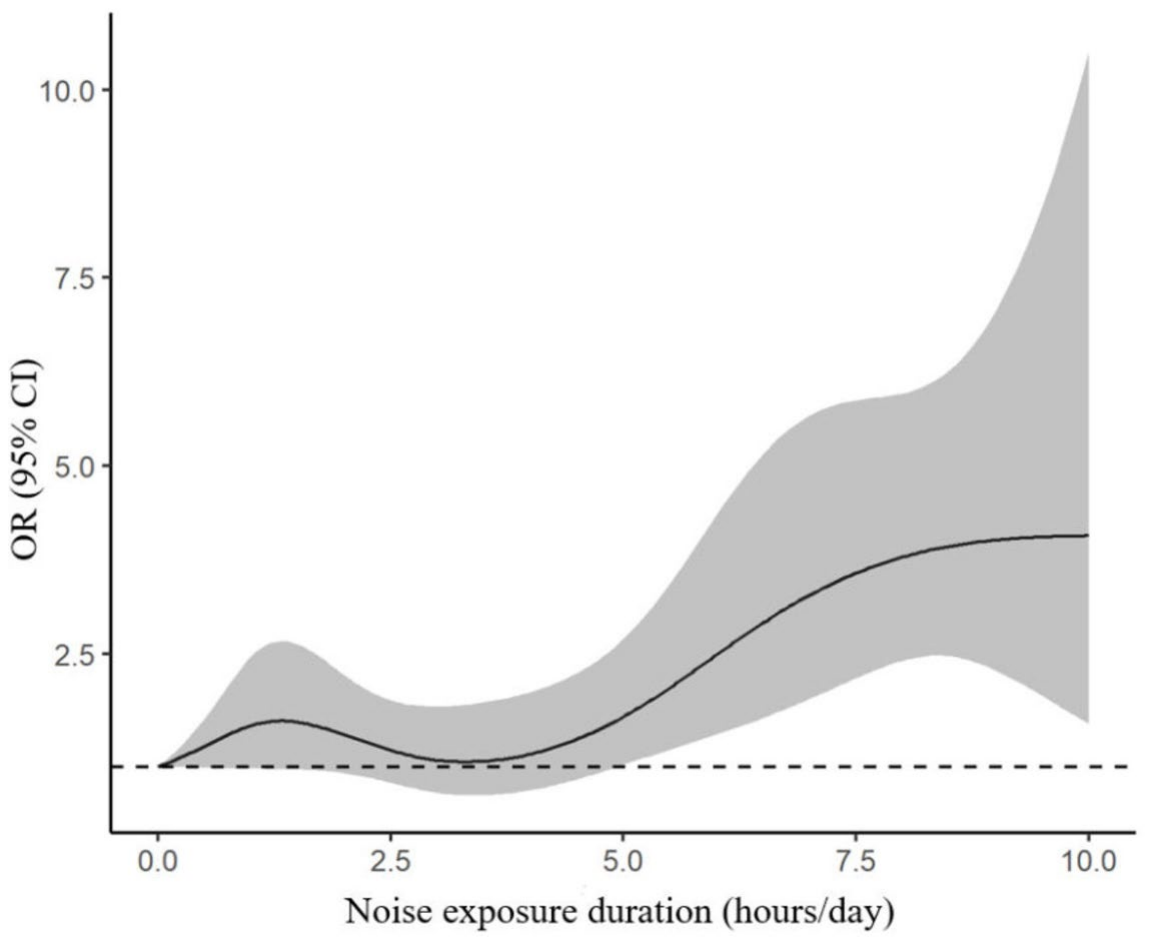
Relationship between noise exposure duration and CHD. Adjusted for age, ethnicity, residence, education, family wealth index, fever in early pregnancy, cold in early pregnancy, taking medicine, gravidity, passive sme, drinking, hair dyeing and perming, cooking, folic acid supplement, and family history of birth defects.

After adjustment for potential confounders, maternal noise exposure duration, when analyzed as a continuous variable, was positively associated with the odds of CHD in offspring (OR, 1.06; 95% CI, 1.03–1.09 per 1-hour/day increase). When exposure duration was categorized into groups, exposure for 4 to ≤8 hours per day was associated with higher odds of CHD (OR, 3.24; 95% CI, 2.02–5.20), and exposure >8 hours per day was also associated with increased odds of CHD (OR, 2.07; 95% CI, 1.19–3.58) compared with no exposure (Table 2). The restricted cubic spline analysis suggested an overall increasing trend between noise exposure duration and CHD risk, although minor fluctuations were observed at lower exposure levels (Figure 2).

Because few participants reported industrial production noise, this category was combined with construction noise. Maternal exposure to transportation noise was associated with elevated CHD risk (OR, 1.75; 95% CI, 1.20–2.56), as was exposure to construction/industrial noise (OR, 2.13; 95% CI, 1.29–3.50) (Table 2). Among participants reporting >8 hours/day of noise exposure, transportation-related noise was the most frequently reported primary source (36/67, 53.7%), followed by construction/industrial noise (17/67, 25.4%) and social activity-related noise (14/67, 20.9%) (Supplementary Table S2).

To further evaluate the role of environmental noise, we analyzed the distance between maternal residences and major traffic arteries or construction sites (Table 3). Analyses showed that living ≤50 meters from a major road was linked to higher CHD risk compared with living >300 meters away (OR, 2.30; 95% CI, 1.66–3.19). Similarly, residing ≤500 meters from a construction site was associated with increased risk compared with distances >1,000 meters (OR, 1.98; 95% CI, 1.37–2.85).

**Table 3:** Relationship between road traffic and construction noise exposure and CHD.

Variables	Cases *N* (%)	Controls *N* (%)	Model 1 crude OR (95% CI), *P* value	Model 2^a^ adjusted OR (95% CI), *P* value	Model 3^b^ adjusted OR (95% CI), *P* value
Distance of residence from major traffic arteries					
>300 meters	261 (44.31)	593 (49.71)	1.00	1.00	1.00
100~ ≤300 meters	94 (15.96)	234 (19.61)	0.91 (0.69, 1.21), 0.523	1.00 (0.74, 1.35), 0.985	0.95 (0.70, 1.29), 0.734
50~ ≤100 meters	112 (19.02)	245 (20.54)	1.04 (0.80, 1.36), 0.781	1.20 (0.90, 1.59), 0.219	1.12 (0.84, 1.51), 0.443
≤50 meters	122 (20.71)	121 (10.14)	2.29 (1.71, 3.06), <0.001	2.38 (1.73, 3.26), <0.001	2.30 (1.66, 3.19), <0.001
Distance of residence and construction sites					
>1000 meters	448 (76.06)	972 (81.47)	1.00	1.00	1.00
500~ ≤1000 meters	65 (11.04)	133 (11.15)	1.06 (0.77, 1.46), 0.717	0.96 (0.68, 1.35), 0.822	0.93 (0.65, 1.32), 0.683
≤500 meters	76 (12.90)	88 (7.38)	1.87 (1.35, 2.60), <0.001	1.95 (1.37, 2.78), <0.001	1.98 (1.37, 2.85), <0.001

^a^Model 2 adjusted age, ethnicity, residence, education, and family wealth index.

^b^Model 3 adjusted age, ethnicity, residence, education, family wealth index, fever in early pregnancy, cold in early pregnancy, taking medicine, gravidity, passive smoke, drinking, hair dyeing and perming, cooking, folic acid supplement, and family history of birth defects.

### Sensitivity analyses

Noise exposure was consistently associated with an increased risk of CHD across all subgroups stratified by maternal age, residence, education level, family wealth index, and gravidity (all *P* < 0.05) (Supplementary Table S3). To further validate the robustness of the findings, propensity scores for noise exposure were calculated for each participant using multivariate logistic regression, and a 1:1 matching process was performed, yielding 471 matched case–control pairs. After matching, the baseline characteristics were well balanced between the case and control groups (Supplementary Table S4). Notably, the association between maternal noise exposure and offspring CHD remained significant after propensity score matching (OR, 1.73; 95% CI, 1.30–2.30) (Supplementary Table S5).

## Discussion

### Principal findings

This multicenter case-control study investigated the association between maternal environmental noise exposure during the periconceptional period and the risk of CHD in offspring in Northwest China. The results demonstrated that maternal environmental noise exposure during the periconceptional period was associated with increased odds of CHD in offspring. A positive exposure–response pattern was observed, with longer reported exposure durations associated with higher odds of CHD. Furthermore, exposure to traffic and construction-related noise was associated with increased odds of CHD after adjustment for a range of individual and family-level confounders. These associations remained robust across various subgroups and sensitivity analyses.

### Comparison with previous literature

To our knowledge, few studies have specifically investigated the association between maternal environmental noise exposure during the periconceptional period and the risk of CHD in offspring. Increasing evidence suggests that environmental exposures during critical periods of embryonic development may contribute to adverse developmental outcomes. However, congenital anomalies represent a heterogeneous group of disorders with distinct embryological origins and developmental mechanisms, and findings from different anomaly subtypes should be interpreted separately.

A meta-analysis by Dzhambov et al. involving 29 studies reported that high levels of prenatal noise exposure were associated with increased risks of congenital anomalies in offspring (22). Several individual studies have further explored specific congenital outcomes. Shi et al. reported an elevated risk of polydactyly among fetuses exposed to maternal noise during pregnancy in a case-control study (23), while Edmonds et al. observed an increased risk of neural tube defects among women residing near airports (24). Although these studies did not directly focus on CHD, they suggest that maternal noise exposure during sensitive developmental periods may influence fetal organogenesis.

Compared with other congenital anomalies, CHD has distinct embryological characteristics, as cardiac development involves complex processes occurring during early embryogenesis. Evidence regarding environmental risk factors for CHD has accumulated for several exposures, particularly ambient air pollution. Previous studies have suggested associations between maternal exposure to traffic-related air pollutants, including PM2.5 and NO₂, and increased CHD risk, highlighting the potential vulnerability of cardiac development to environmental stressors. However, whether maternal environmental noise exposure independently contributes to CHD risk remains insufficiently understood. Gong et al. also noted an increased CHD risk in a cohort of noise-exposed pregnant women, though the study lacked fully adjusted results (25).

Our study extends previous evidence by focusing specifically on maternal environmental noise exposure during the periconceptional period and CHD risk in offspring. The relatively large sample size, adjustment for a wide range of individual and family-level potential confounders, and multiple sensitivity analyses strengthen the robustness of our findings and provide additional epidemiological evidence regarding this underexplored environmental exposure.

In addition, experimental studies provide biological plausibility for potential adverse effects of maternal noise exposure on fetal development. Experiments in guinea pigs and sheep have demonstrated that prenatal noise exposure may impair offspring hearing development (26, 27). Although some animal studies have not identified specific associations with congenital malformations, others have reported increased risks of fetal death or impaired embryonic development following high-level noise exposure (15). These findings suggest that maternal noise exposure may influence fetal development through complex biological pathways, although the mechanisms underlying potential effects on cardiac development require further investigation.

### Impact of traffic and construction noise on CHD risk

Our study found that maternal exposure to traffic and construction noise during periconceptional period was associated with an elevated risk of CHD in offspring. The observed associations with residential proximity to major traffic arteries and construction sites further support the potential relevance of these environmental noise sources. Previous research has linked traffic noise to various adverse pregnancy outcomes. For instance, exposure to traffic noise has been associated with an increased risk of orofacial clefts in offspring (28). Smith et al. reported a positive association between road traffic noise and perinatal mortality in a cross-sectional study (29). A meta-analysis also identified significant associations between traffic noise and adverse birth outcomes, including low birth weight, preterm birth, and small for gestational age (10). In a national cohort study, Selander et al. reported an association between maternal occupational noise exposure and offspring hearing impairment, suggesting that prenatal noise exposure may influence fetal developmental processes beyond traditional birth outcomes (30). Additionally, Dzhambov et al. reported associations between residential and occupational noise exposure and adverse developmental outcomes, including congenital malformations and small-for-gestational-age births (22).

Due to the small number of participants exposed to industrial production noise, industrial production noise was combined with construction noise for analysis. To the best of our knowledge, no previous studies have specifically examined the relationship between maternal exposure to traffic and construction noise and the risk of CHD in offspring. This novel finding warrants further investigation and may have important implications for public health and urban planning.

### Potential mechanisms linking noise exposure to CHD

The biological mechanisms underlying the association between maternal noise exposure during the periconceptional period and the development of CHD in offspring are likely multifactorial and complex. Current evidence suggests that noise acts as a physiological stressor, activating both the sympathetic-adrenal-medullary (SAM) system and the hypothalamic-pituitary-adrenal (HPA) axis (31, 32). This activation results in the release of neurohormones and elevated levels of stress-related hormones, such as cortisol and catecholamines, which may disrupt critical processes in periconceptional embryogenesis and fetal organogenesis (33, 34). Moreover, previous studies have reported associations between maternal exposure to psychosocial stress and an increased risk of congenital anomalies in offspring (35). These findings support the hypothesis that maternal stress responses—whether triggered by psychological factors or environmental stressors such as noise—can negatively affect fetal development.

Given the limited body of research specifically addressing the link between noise exposure and CHD, further studies are needed to elucidate the biological pathways involved. Investigations focusing on the timing, duration, and intensity of noise exposure during early pregnancy may provide insights into critical windows of vulnerability in cardiac morphogenesis.

### Strengths and limitations

This multicenter case-control study systematically examined the association between maternal periconceptional noise exposure and the risk of CHD in offspring, providing important evidence regarding an underexplored environmental exposure associated with CHD. Several strengths enhance the robustness of our findings, including the large sample size, adjustment for a wide range of individual and family-level potential confounders, and multiple sensitivity analyses that consistently supported the main results.

However, several limitations should be considered. First, although the periconceptional period was selected as a biologically plausible exposure window, we could not determine whether the observed associations reflected effects specific to this period or cumulative exposure patterns before conception and throughout pregnancy. In addition, exposure changes after pregnancy recognition or at specific gestational stages could not be evaluated. These findings should be interpreted within the context of the periconceptional exposure window. Second, maternal noise exposure was assessed using self-reported questionnaire data, which may introduce exposure misclassification and recall bias. Self-reported exposure was not validated against objective noise measurements or modeled exposure estimates. Individual differences in noise sensitivity, annoyance thresholds, and undiagnosed hearing impairment could not be completely excluded. Moreover, objective noise measurements and geospatial exposure data were unavailable, preventing estimation of quantitative noise levels based on dB (A), classification according to regulatory noise zones, or objective validation of residential proximity to major roads and construction sites. Detailed information on residential acoustic characteristics, personal activity patterns, and specific exposure contexts (e.g., occupational versus residential environments) was also unavailable. Third, residual confounding by other environmental and neighborhood-level factors cannot be completely excluded. Although individual and household-level factors were adjusted, outdoor air pollution (e.g., PM2.5 and NO₂) and neighborhood-level socioeconomic characteristics were not available in the current dataset. Finally, the hospital-based case-control design may limit the generalizability of our findings despite the use of stringent inclusion criteria and frequency matching.

Despite these limitations, this study provides important evidence regarding the potential association between maternal environmental noise exposure and CHD risk. Future research integrating objective exposure assessment and longitudinal study designs will help further clarify the relationship between environmental noise exposure and fetal cardiac development. Such evidence may contribute to the development of targeted public health strategies and urban planning interventions aimed at reducing prenatal environmental noise exposure and improving maternal and child health outcomes.

## Conclusion

In this multicenter case-control study, maternal exposure to environmental noise during the periconceptional period was associated with increased odds of CHD in offspring, with a positive exposure–response pattern observed for exposure duration. Traffic and construction-related noise were also associated with increased odds of CHD after adjustment for individual and family-level confounders. These findings highlight environmental noise as a potentially modifiable prenatal risk factor. From a maternal and child health perspective, reducing environmental noise exposure during the periconceptional period may have implications for future CHD prevention strategies and should be considered in public health and urban planning initiatives.

## Data Availability

The raw data supporting the conclusions of this article will be made available by the authors, without undue reservation.
