# Supplementary material for: Maternal environmental noise exposure during the periconceptional period and risk of congenital heart disease in offspring: a case-control study in Northwest China
Source: Front Public Health. 2026 Sep 1;14:1923108. doi: 10.3389/fpubh.2026.1923108 (PMC13574559; doi:10.3389/fpubh.2026.1923108)
Supplement: Supplementary file 1 [file Table_1.docx]

**Maternal Environmental Noise Exposure in Early Pregnancy and Risk of Congenital Heart Disease: A Case-Control Study in Northwest China**

Mingxin Yan ^1,2†^, Yan Zhao ^3†^, Yubo Zhang ^1^, Doudou Zhao ^4^, Bin Wang ^2^, Jianting Si ^2^, Yuanhan Sun ^2^, Guoqiang Dong ^2^, Leilei Pei ^1,*^ and Pengfei Qu ^4**^

^1^ Department of Epidemiology and Biostatistics, School of Public Health, Xi’an Jiaotong University Health Science Center, Xi’an, China.

^2^ Center for Occupational Safety and Health, Institute for Hygiene of Ordnance Industry, Xi’an, China.

^3^ Department of Dermatology, The First Affiliated Hospital of Xi’an Jiaotong University, Xi’an, China.

^4^ Translational Medicine Center, Northwest Women’s and Children’s Hospital, Xi’an, China.

*Corresponding author: Leilei Pei, PhD

Email: [pll_paper@126.com](mailto:pll_paper@126.com)

**Corresponding author: Pengfei Qu, PhD

Email: xinxi3057@163.com

†These authors have contributed equally to this work

**Supplementary Table 1** Association of noise exposure with different CHD subtypes.

|  |  | Noise exposure, N (%) | | Model 1 | Model 2^a^ | Model 3^b^ |
| --- | --- | --- | --- | --- | --- | --- |
|  |  | Yes | No | Crude OR (95%CI) | Adjusted OR (95%CI) | Adjusted OR (95%CI) |
| Main CHD types |  |  |  |  |  |  |
| VSD |  | 39 (21.08) | 146 (78.92) | 1.34(0.92,1.96) | 1.30(0.88,1.93) | 1.19(0.79,1.77) |
| ASD |  | 17(23.94) | 54(76.06) | 1.56(0.89,2.73) | 1.56(0.88,2.74) | 1.57(0.88,2.80) |
| PDA |  | 16(21.62) | 58(78.38) | 1.36(0.77,2.40) | 1.37(0.78,2.43) | 1.36(0.76,2.44) |
| AVSD |  | 9(18.00) | 41(82.00) | 1.07(0.51,2.22) | 1.03(0.49,2.15) | 0.94(0.44,2.00) |
| TOF |  | 16(37.21) | 27(62.79) | 2.99(1.59,5.61) | 3.11(1.62,5.95) | 2.85(1.46,5.56) |

VSD: ventricular septal defect; ASD: atrial septal defect; PDA: patent ductus arteriosus; AVSD: atrioventricular septal defect; TOF: tetralogy of Fallot.

Multiple testing correction was performed using the Bonferroni method for CHD subtype analyses.

^a^ Model 2 adjusted age, ethnicity, residence, education, and family wealth index.

^b^ Model 3 adjusted age, ethnicity, residence, education, family wealth index, fever in early pregnancy, cold in early pregnancy, taking medicine, gravidity, passive smoke, drinking, hair dyeing and perming, cooking, folic acid supplement, and family history of birth defects.

**Supplementary Table 2** Distribution of primary reported noise sources among participants with >8 hours/day noise exposure

| Primary reported noise source among participants with >8 hours/day exposure | Cases, n (%) | Controls, n (%) | Total, n (%) |
| --- | --- | --- | --- |
| Transportation | 16 (50.00) | 20 (57.14) | 36 (53.73) |
| Construction/industrial production | 10 (31.25) | 7 (20.00) | 17 (25.37) |
| Social life | 6 (18.75) | 8 (22.86) | 14 (20.90) |
| Total | 32 (100.00) | 35 (100.00) | 67 (100.00) |

**Supplementary Table 3** Association between noise exposure and CHD in subgroups.

| Subgroups | Cases  （N=589） | Controls  （N=1193） | *P* value | Adjusted OR (95%CI) ^a^ |
| --- | --- | --- | --- | --- |
| Age, n (%) |  |  |  |  |
| <30 years | 371(62.99) | 802(67.23) | 0.004 | 1.64(1.17, 2.31) |
| ≥30 years | 218(37.01) | 391(32.77) | <0.001 | 2.56(1.61, 4.05) |
| Residence, n (%) |  |  |  |  |
| Urban | 196(33.28) | 796(66.72) | <0.001 | 2.13(1.45, 3.13) |
| Rural | 393(66.72) | 397(33.28) | 0.003 | 1.81(1.22, 2.67) |
| Education, n (%) |  |  |  |  |
| Senior high school or lower | 261(44.31) | 270(22.63) | <0.001 | 1.86(1.34, 2.58) |
| College or above | 328(55.69) | 923(77.37) | 0.002 | 2.27(1.36, 3.79) |
| Family wealth index, n (%) |  |  |  |  |
| Poor | 308(52.29) | 328(27.49) | 0.002 | 2.13(1.33, 3.41) |
| Moderate | 165(28.01) | 395(33.11) | 0.034 | 1.69(1.04, 2.74) |
| Rich | 116(19.70) | 470(39.40) | 0.001 | 2.21(1.36, 3.58) |
| Gravidity, n (%) |  |  |  |  |
| 1 | 230(39.05) | 617(51.72) | 0.004 | 1.85(1.22, 2.81) |
| ≥2 | 359(60.95) | 576(48.28) | <0.001 | 2.01(1.40, 2.88) |

^a^ Adjusted for covariates other than subgroup variables, with noiseless exposure as the reference.

**Supplementary Table 4** Baseline characteristics of the case and control groups after propensity score matching.

| Variables | Cases  （N=471） | Controls  （N=471） | *χ^2^/Z* | *P* value |
| --- | --- | --- | --- | --- |
| Age, median (IQR) | 28.27(25.50，30.70) | 27.31(24.47，31.02) | -1.659 | 0.098^a^ |
| Ethnicity, n (%) |  |  | 0.092 | 0.762 |
| Han | 465(98.73) | 466(98.94) |  |  |
| Other | 6(1.27) | 5(1.06) |  |  |
| Residence, n (%) |  |  | 0.157 | 0.692 |
| Urban | 194(41.19) | 200(42.46) |  |  |
| Rural | 277(58.81) | 271(57.54) |  |  |
| Education, n (%) |  |  | 0.220 | 0.639 |
| Senior high school or lower | 184(39.07) | 177(37.58) |  |  |
| College or above | 287(60.93) | 294(62.42) |  |  |
| Family wealth index, n (%) |  |  | 0.092 | 0.955 |
| Poor | 206(43.74) | 210(44.59) |  |  |
| Moderate | 151(32.06) | 147(31.21) |  |  |
| Rich | 114(24.20) | 114(24.20) |  |  |
| Fever in early pregnancy, n (%) |  |  | 0.060 | 0.806 |
| No | 434(92.14) | 436(92.57) |  |  |
| Yes | 37(7.86) | 35(7.43) |  |  |
| Cold in early pregnancy, n (%) |  |  | 0.022 | 0.882 |
| No | 348(73.89) | 346(73.46) |  |  |
| Yes | 123(26.11) | 125(26.54) |  |  |
| Taking medicine, n (%) |  |  | 0.237 | 0.626 |
| No | 373(79.19) | 379(80.47) |  |  |
| Yes | 98(20.81) | 92(19.53) |  |  |
| Gravidity, n (%) |  |  | 0.039 | 0.843 |
| 1 | 200(42.46) | 203(43.10) |  |  |
| ≥2 | 271(57.54) | 268(56.90) |  |  |
| Passive smoke, n (%) |  |  | 0.017 | 0.896 |
| No | 220(46.71) | 222(47.13) |  |  |
| Yes | 251(53.29) | 249(52.87) |  |  |
| Drinking, n (%) |  |  | 1.351 | 0.245 |
| No | 463(98.30) | 467(99.15) |  |  |
| Yes | 8(1.70) | 4(0.85) |  |  |
| Hair dyeing and perming, n (%) |  |  | 0.122 | 0.727 |
| No | 455(96.60) | 453(96.18) |  |  |
| Yes | 16(3.40) | 18(3.82) |  |  |
| Cooking, n (%) |  |  | 0.181 | 0.670 |
| No | 139(29.51) | 145(30.79) |  |  |
| Yes | 332(70.49) | 326(69.21) |  |  |
| Folic acid supplement, n (%) |  |  | 0.464 | 0.496 |
| No | 80(16.99) | 88(18.68) |  |  |
| Yes | 391(83.01) | 383(81.32) |  |  |
| Family history of birth defects, n (%) |  |  | 0.227 | 0.634 |
| No | 463(98.30) | 461(97.88) |  |  |
| Yes | 8(1.70) | 10(2.12) |  |  |

IQR, interquartile range.

^a^ Wilcoxon rank test.

**Supplementary Table 5** Association between noise exposure and CHD after propensity scores matching.

|  | Noise exposure, n (%) | |  | OR (95%CI), *P* value |
| --- | --- | --- | --- | --- |
|  | Yes | No |  |  |
| Controls | 74 (15.71) | 397 (84.29) |  | 1.00 |
| Cases | 127 (26.96) | 344 (73.04) |  | 1.73 (1.30, 2.30), <0.001 |
